# *In silico* analysis of onion chitinases using transcriptome data

**DOI:** 10.6026/97320630014440

**Published:** 2018-08-31

**Authors:** Rupesh Kumar Mohapatra, Satyabrata Nanda

**Affiliations:** 1 Center for Biotechnology, Siksha 'O' Anusandhan University, Bhubaneswar, Odisha 751003, India; 2 State Key Laboratory of Rice Biology, China National Rice Research Institute, Hangzhou, Zhejiang 311440, China

**Keywords:** Chitinases, Glycoside hydrolases (GH), Motifs, Phylogenetic analysis, *Allium cepa*

## Abstract

Chitinases are glycoside hydrolase (GH) family of proteins having multifaceted roles in plants. It is of interest to identify and characterize chitinase-encoding genes from the popular bulbous plant onion (*Allium cepa* L.). We have used the EST sequences for onion chitinases to elucidate its functional features using sequence, structure and functional analysis. These contigs belong to the GH19 chitinases family according to domain architecture analysis. They have highly conserved chitinase motifs including motifs exclusive to plant chitinases as implied using the MEME based structural characterization. Estimation of biochemical properties suggested that these proteins have features to form stable and hydrophilic proteins capable of localizing extracellular and in vacuoles. Further, they have multiple cellular processes including defense role as inferred by DeepGO function prediction. Phylogenetic analysis grouped them as class I and class VII plant chitinase, with possible abundance of class I chitinase in onion. These observations help in the isolation and functional validation of onion chitinases.

## Background

Onion (Allium cepa L.) is an important and most widely cultivated bulbous vegetable with great commercial and medicinal significance [1]. India is the second largest producer and the largest exporter of onions, producing more than 19 million tons and exporting more than 1 million ton per year [2]. On one hand the demand for onion is rising and on the other hand the vegetable faces huge crop losses due to pathogen infections majorly from fungal pathogens [3]. Plant chitinases (EC 3.2.1.14) are from pathogenesis-related (PR) protein family having chitin as the substrate target, which is usually the prime component of a phyto-pathogen [4]. Chitinases have been reported to be involved in response to an array of stimuli including mechanical injury, phyto-hormones, temperature alteration, salinity, metal stress, and pathogen invasion [5, 6, 7, 8]. While, diverse functionality is linked to plant chitinases, based on their sequence conservations of their catalytic domain and mechanism of action, they are classified into two glycoside hydrolase (GH) (GH18 and GH19) families [9]. No sequence or structural similarities between GH18/GH19 indicates the independent evolution of chitinase family. Even though, GH18 family includes some plant chitinases, but mainly consists of chitinases from animals, fungi, bacteria and viruses. On the contrary, most plant chitinases belong to GH19 family along with a few chitinases from nematodes and bacteria [10]. Chitinases in plants are divided into seven classes (class I-VII), which belong to both GH18 and GH19 families [4]. The functions and localization of different class chitinases differ from one another; for instance, class I chitinase are basic in nature and localize in vacuole, whereas class II are acidic in nature and localize extracellularly [11].

The availability of EST data in public databases like dbEST facilitates the mining, prediction and characterization of candidate genes by computational biological methods. Several genes having vital functional attributes in processes like seed development [12], plant growth [13], and defense response [14, 15], microsatellites [16], and micro-RNAs [17] have been identified using mining of dbESTs and genome survey sequences (dbGSS) sequences. In the current work, an EST mining-based identification of chitinases in A. cepa has been carried out using already reported plant chitinase sequences as bait. Further insilico analysis of the identified highly homologous contigs revealed the structural organization and domain architecture of the identified onion chitinases. Functional annotations and biochemical properties of the identified onion chitinases were predicted using several bioinformatics tools. Finally, based on the predicted structural information along with phylogenetic classification the identified onion chitinases are divided into respective chitinase classes.

## Methodology

### EST dataset of onion chitinases

The NCBI public database dbEST contains single-pass cDNA or expressed sequence tag sequences from animals, plants, and microorganisms. A total of 20255 EST sequences expressed in different physiological conditions in different tissues deposited in dbEST were downloaded in FASTA format. All 20255 ESTs were screened against the UniVec database of NCBI [18] to detect vector and adapter sequence contaminations, and such detected sequences were subsequently removed. Obtained clean reads with no sequence contaminations were subsequently fed using CAP3 sequence assembly program [19] to generate a nonredundant dataset of contigs.

### Sequence homologs of onion chitinases

The Basic Local Alignment Search Tool (BLAST) variant TBLASTN was used to perform reverse alignment of selected previously reported chitinases on A. cepa contigs. All chitinase clusters found in A. cepa database were translated to obtain their protein sequences. The open reading frames (ORFs) for each searched contig was obtained using ExPASy translate tool [20]. Protein sequences obtained were used for second round of BLASTp search against the non-redundant protein database at NCBI to identify their closest homologs. Multiple alignments of proteins deduced from the selected contig sequences were performed using Clustal Omega program [21].

### De novo motifs and domain architecture

De novo motif predictions and motif elicitation of the selected contigs were performed using Multiple Expectation Maximization for motif Elicitation (MEME) tool [22]. The motif searches were performed for zero or one occurrence per sequence to restrict the number of statistically overrepresented motifs in the dataset. Default width of MEME motif searches were employed having a minimum and maximum motif width of 6 and 50, respectively. Additional domains were detected using the Simple Modular Architecture Research Tool (SMART) program [23]. The protein folding states of the identified onion chitinases were predicted using the FoldIndex program [24].

### Estimation of biochemical parameters

Prediction of various peptide properties like molecular weight (Mw) and isoelectric points (pI) of the selected onion contigs were achieved using Compute pI/Mw [25]. Peptide properties including amino acid composition, instability index, aliphatic index, and grand average of hydropathicity (GRAVY) were predicted by using Protparam tool [26]. Subcellular localization of the onion contigs was performed using mGOASVM (Plant V2) server [27].

### Phylogenetic analysis

Multiple sequence alignment of the selected nine onion contigs was performed using the MUSCLE program [28] keeping all parameters to default. Phylogenetic analysis was performed using the aligned contigs and other plant chitinase sequences by employing the neighbor-joining (NJ) method of phylogenetic classification with Poisson correction, 1000 bootstrap replicates and pairwise deletion by using the Molecular Evolutionary Genetic Analysis (MEGA v 7) package [29].

## Results and Discussion

**Figure 1:**
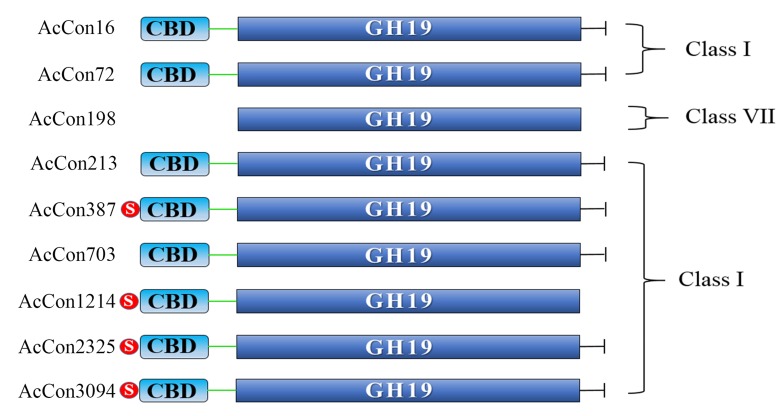
Domain architecture of the A. cepa chitinases. S: signal peptide; CBD: Chitin binding domain; GH19: glycoside hydrolase family 19 domain; -|: C-terminal catalytic domain.

Assembling the cleaned EST sequences by using CAP3 sequence assembly program resulted in a total of 4175 contigs. Reverse alignment on the generated contigs were done using TBLASTN with previously reported plant chitinases. The bait chitinase sequences were taken from two widely used model plants Arabidopsis thaliana and Oryza sativa chitinases as listed by Grover [30], comprising of both GH18 and GH19 family chitinases. Sequence homology assessment by consecutive rounds of BLAST searches resulted in identification of nine (AcCon16, AcCon72, AcCon198, AcCon213, AcCon387, AcCon703, AcCon1214, AcCon2325, and AcCon3094) highly homologous onion contigs with previously reported plant chitinases (Table 1). All nine contigs were found to carry the GH19 domain, thus, identified as GH19 family chitinases. No member of GH18 chitinase was identified in our in silico approach of onion chitinase identification. In Angiosperms, GH19 family chitinases are seen in abundance and their distribution is localized to higher plants. On the contrary, GH19 family chitinases are rarely seen in microorganisms like bacteria, and are completely absent in archaea [10]. Further structural characterization of the onion chitinase by Prosite and SMART domain analysis resulted in identification of the additional domains like chitin binding domain (CBD) and signal peptides. The domain architecture of nine onion contigs were then deduced according to the information obtained after the multiple domain scans (Figure 1).

**Figure 2:**
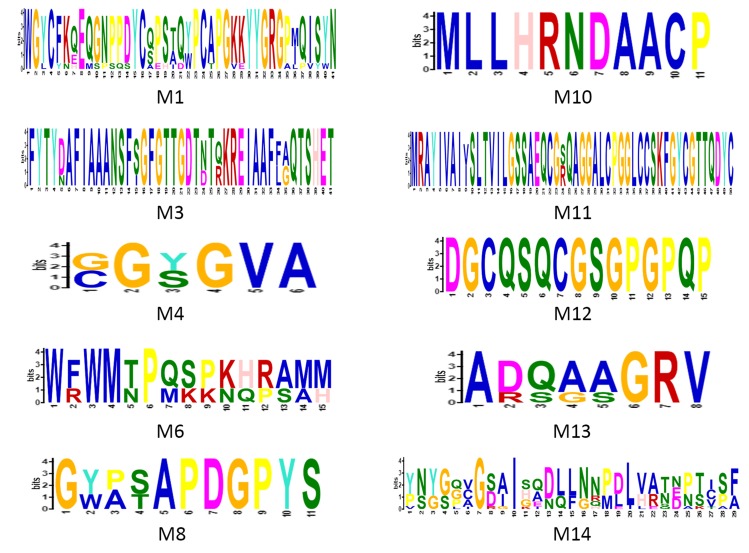
The conserved sequence motifs (represented as weblogos) possessed by the A. cepa chitinases. Letter M and the corresponding Arabic numerical represent the motif number as conserved in plants and other chitinases.

De novo motif prediction from the selected onion contig sequences using MEME tool revealed the presence of several conserved motifs at both N-terminal and C-terminal regions (Figure 2). A highly conserved motif across the GH19 family chitinases "M1" [31], having signature sequence "Y[YF]GRGPIQ[ST][WY]N" was found to be present in all nine onion chitinases. M1 motif has been reported to form a substrate binding cleft during its activity in plants [31]. Additionally, 9 more structurally conserved motifs in GH19 chitinases were discovered from the selected onion contig sequences. Motif M3, M4, and M6 are conserved in chitinases found in purple bacteria, actinobacteria, and plants [10]. Furthermore, M8, M10, M11, M12, and M13, found in the onion chitinases are exclusively present in plant GH19 chitinases. Thus, the presence of highly conserved GH19 structural motifs and exclusive plant GH19 chitinase motifs strongly support that all nine onion chitinases to be possible functional chitinases.

Nine onion contigs with high sequence homology to previously reported plant chitinases contains conserved canonical GH19 chitinase motifs were identified in our study. Their predicted peptide length varied from 164 (AcCon3094) to 240 (AcCon387 and AcCon1214) amino acids, whereas their predicted molecular weight ranged from 17.28 to 25.53 kDa. Predicted proteins forms of all nine contigs were found to be stable having less than 40- instability index (except AcCon198) and higher aliphatic indexes ranging from 42.33 to 72.70 (Table 2). Nature of hydropathy prediction of these nine chitinases revealed that all of them showed a negative GRAVY value indicating to be hydrophilic in nature.

Ontology predictions of all nine-onion chitinases were performed using DeepGo analysis [32]. DeepGO predicts the function of a protein from its sequence by employing an algorithm that utilizes the dependencies of the gene ontology (GO) classes as background information to construct a deep learning model. Prediction of functions of all nine contigs revealed their possible catalytic and hydrolase activities, which are the key functions of a plant chitinase (Table 2). In addition, the biological function prediction of all 9-onion chitinases revealed that all of them potentially participate in cellular or multi-organism processes. However, AcCon198, AcCon387, AcCon1214, and AcCon2325 were predicted with a function of response to stimulus, which suggest their possible role in onion defense responses. These predicted functional attributes of the contigs strengthens the assumption that the identified contigs to be functional chitinases.

Chitinases are a diverse group of enzymes having different enzymatic activities in different parts of a plant and have diverse cellular localizations. We predicted the sub-cellular localization of the identified onion chitinases using the mGOASVM server. mGOASVM prediction accuracy of the subcellular locations are significantly higher than the conventional methods of subcellular-localization prediction using tools like TargetP, SignalP, or even iLoc-Plant [27]. Results from mGOASVM server prediction revealed that AcCon16, AcCon213, and AcCon703 localize in vacuole, whereas AcCon72, AcCon198, AcCon387, AcCon1214, AcCon2325, and AcCon3094 were secretory chitinases.

**Figure 4:**
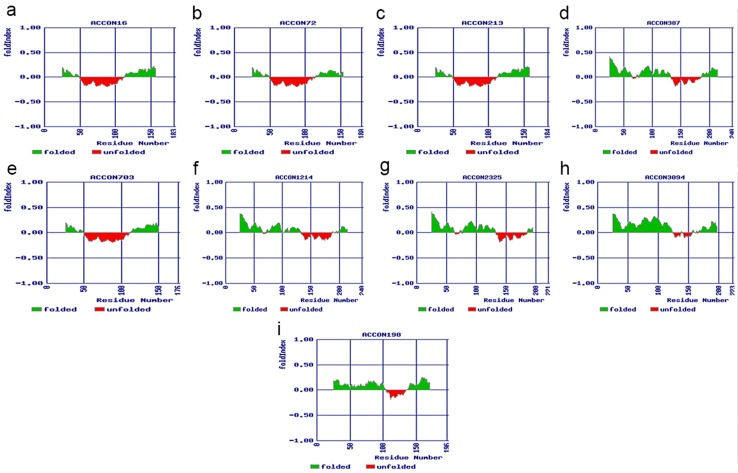
FoldIndex plot for the A. cepa chitinases. a - h: class I chitinases, i: class VII chitinase. The positive and values on y-axis represents ordered and disordered peptides, respectively. Green on the graph indicates amino acids being ordered, red represents disordered amino acids.

As the phylogenetic analysis confirmed that the chitinases are of class I and class VII, we tried to predict their protein folding stages using the FoldIndex program. The folding states of all nine-onion chitinases were predicted (Figure 4). FoldIndex estimates the folding of a given protein sequence based on the net charge and average hydrophobicity of the input sequence [24]. The onion chitinases exhibited different predicted folding properties. AcCon16, AcCon72, AcCon213, AcCon387, AcCon703, AcCon1214, and AcCon2325 contained higher percentage of disordered residues, whereas AcCon198 and AcCon3094 showed small unfolding and least disordered sequences. The results obtained were in accordance with Mishra et al. [11] who reported that class I chitinases possess more disordered sequences than others.

## Conclusion

It is of interest to perform a comprehensive structural evaluation of onion chitinases using various computational approaches. We have found nine highly homologous onion contigs with other plant chitinases having conserved motifs. Further, their domain architecture contains well-conserved GH19 domain in addition to CBD structural and signal peptides. DeepGo function prediction suggests that four onion chitinases have defense response. Phylogenetic classification confirmed that the onion chitinases belong to class I and class VII. These observations serve as a framework for the future characterization and functional assessment of onion chitinases. Moreover, it adds insights to the understanding of the distribution and diversity of onion chitinases.

## Figures and Tables

**Table 1:** Homologs of A. cepa chitinases

Contigs	Homologous Sequence	Source	Identity	Accession no.	E value
AcCon16	Chitinase	Allium sativum	92%	AAA32641	3.00E-122
AcCon72	Chitinase 1	Lilium longiflorum	82%	AIR76996	3.00E-103
AcCon198	Chitinase 7	Saccharum sp.	74%	AIN36552	2.00E-101
AcCon213	Chitinase 1-like	Ananas comosus	82%	XP_020079844	2.00E-144
AcCon387	Endochitinase	Triticum aestivum	75%	AHY24795	1.00E-114
AcCon703	Chitinase	Poa pratensis	82%	AAF04454	4.00E-103
AcCon1214	Endochitinase	Persea americana	78%	CAB01591	3.00E-122
AcCon2325	Predicted: Chitinase 1	Elaeis guineensis	76%	XP_010941404	3.00E-117
AcCon3094	chitinase Ib	Chimonanthus praecox	72%	ACN55075.1	2.00E-60

**Table 2:** Biochemical and functional features of A. cepa chitinases

Name	pI value	MW	Instability Index	Aliphatic Index	GRAVY Value	Localization	Molecular function	Biological process
AcCon16	6.02	19.97	35.55	44.18	-0.533	Vacuole	Catalytic activity	Multi-organism process (GO:0051704)
							(GO:0003824)	
AcCon72	6.39	19.74	34.28	45.17	-0.623	Secretory	Catalytic activity	Multi-organism process (GO:0051704)
							(GO:0003824)	Cellular process (GO:0009987)
AcCon198	5.24	21.75	40.75	72.7	-0.222	Secretory	Hydrolase activity	Response to stimulus (GO:0050896)
							(GO:0016787)	Single-organism process (GO:0044699)
								Cellular process (GO:0009987)
AcCon213	6.01	20.01	35.66	44.26	-0.518	Vacuole	Catalytic activity	Multi-organism process (GO:0051704)
							(GO:0003824)	Cellular process (GO:0009987)
AcCon387	5.53	25.37	29.35	51.75	-0.306	Secretory	Catalytic activity	Response to stimulus (GO:0050896)
							(GO:0003824)	Cellular process (GO:0009987)
AcCon703	6.87	19.24	39.81	42.33	-0.58	Vacuole	Hydrolase activity	Cellular process (GO:0009987)
							(GO:0016787)	Single-organism process (GO:0044699)
AcCon1214	8.58	25.53	30.04	50.54	-0.364	Secretory	Hydrolase activity	Cellular process (GO:0009987)
							(GO:0016787)	Single-organism process (GO:0044699)
								Response to stimulus (GO:0050896)
AcCon2325	4.87	23.07	26.03	53.98	-0.29	Secretory	Catalytic activity	Response to stimulus (GO:0050896)
							(GO:0003824)	Cellular process (GO:0009987)
AcCon3094	8.63	17.28	28.57	60.79	-0.141	Secretory	Catalytic activity	Single-organism process (GO:0044699)
							(GO:0003824)	Cellular process (GO:0009987)

**Figure 3:**
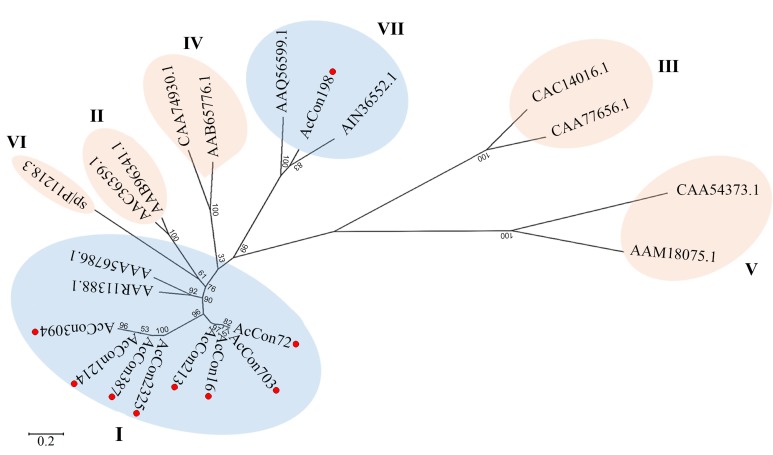
Phylogenetic tree constructed using neighbor-joining method using MEGA 7.0. Each class of chitinases (class I-VII) is shaded together. Red dot denotes the A. cepa contigs. Roman letters indicate different class of chitinases. The percentage of replicate trees in which the associated taxa clustered together in the bootstrap test (1000 replicates) is shown next to the branches. Accessions used in constructing the tree were taken from NCBI representing their corresponding class of chitinases.

## References

[1] Nanda S (2016). Plant Pathol J..

[2] FAOSTAT (2017). Food Agriculture Organization Statistics Division..

[3] Kareem MA (2012). Bioinfolet..

[4] Su Y (2015). Sci Rep..

[5] Goñi O (2009). J Plant Physiol..

[6] Kumar V (2009). Planta..

[7] Liliane LB (2004). Genet Mol Biol..

[8] Liu B (2010). Mol Biol Rep..

[9] Fukamizo T. (2000). Curr Protein Pept Sci..

[10] Udaya Prakash NA (2010). J Mol Evol..

[11] Mishra AK (2015). Indian J Biochem Biophys..

[12] Emilia NS (2015). African J Biotechnol..

[13] Kar B (2012). Bioinformation..

[14] Joshi RK (2011). Bioinformation..

[15] Joshi RK (2011). Bioinformation..

[16] Chand SK (2015). Bioinformation..

[17] Baghban KB (2017). Interdiscip Sci Comput Life Sci..

[18] Huang X, Madan A. (1999). Genome Res..

[19] ftp://ftp.ncbi.nlm.nih.gov/pub/UniVec/.

[20] http://web.expasy.org/translate/.

[21] https://www.ebi.ac.uk/Tools/msa/clustalo/.

[22] Bailey TL (2006). Nucleic Acids Res..

[23] Letunic I (2015). Nucleic Acids Res..

[24] Prilusky J (2005). Bioinformatics..

[25] Wilkins MR (1999). Methods Mol Biol..

[26] Artimo P (2012). Nucleic Acids Res..

[27] Wan S (2012). BMC Bioinformatics..

[28] Edgar R. (2004). Nucleic Acids Res..

[29] Kumar S (2016). Mol Biol Evol..

[30] Grover A. (2012). Crit Rev Plant Sci..

[31] Huet J (2008). Biochemistry..

[32] Kulmanov M (2018). Bioinformatics..

[33] Li DM (2009). Plant Mol Biol Report..

